# Reproductive and Developmental Toxicity of Human Umbilical Cord Blood Mononuclear Cells

**DOI:** 10.3390/biomedicines14030508

**Published:** 2026-02-25

**Authors:** Zhanna Dzampaeva, Sergey Skupnevskii, Rodion Saveljev, Yana Morozova, Sergey Radaev, Vladimir Smirnov, Andrey Grin

**Affiliations:** 1 Laboratory of System Ecological Analysis, North Ossetian State University, 362025 Vladikavkaz, Russia; 2 N.V. Sklifosovsky Research Institute of Emergency Medicine, Moscow Healthcare Department, 129010 Moscow, Russia; 3 E.I. Chazov National Medical Research Center of Cardiology of Healthcare Ministry of the Russian Federation, 121552 Moscow, Russia

**Keywords:** developmental toxicity, mononuclear cells, umbilical cord, reproductive toxicity, rats

## Abstract

**Background/Objectives**: The attention of world science has been focused on human umbilical cord blood cell (hUCB) products for the treatment of various human diseases. The prospects for using hUCB stem from the availability of the material, non-invasive collection procedure, low immunogenicity, multipotency and non-tumorigenicity. But information about the acute toxicity, reproductive and developmental toxicity of hUCB mononuclear cells (MNCs) remains insufficient. Thus, the aim of this study is to assess the reproductive and developmental toxicity of human umbilical cord blood mononuclear cells on Wistar rats. **Methods**: In the fertility and early embryonic development study, human umbilical cord blood mononuclear cells (hUCB-MNCs) were administered at dose levels of 4.28 × 10^8^ cells/kg and 8.57 × 10^8^ cells/kg to male and female rats during the pre-mating, mating and gestation period. In the embryo–fetal development study, the pregnant female rats also received hUC-MNCs at doses of 4.28 × 10^8^ cells/kg and 8.57 × 10^8^ cells/kg. **Results**: In gestational data, including fertility rate, pregnancy rate, corpora lutea and implantation sites counts, dead and absorption fetuses’ number, body weight and craniocaudal size of fetuses, anomalies in fetal development showed no statistically significant changes in 4.28 × 10^8^ cells/kg (low dose) and 8.57 × 10^8^ cells/kg (high dose) dose groups of hUCB-MNCs to negative control group. External, visceral and skeletal examination of the fetuses in all experimental groups also showed no changes. Embryo–fetal development study in low and high groups of hUCB-MNCs application also showed no changes in the negative control group. **Conclusions**: This reproductive and developmental toxicity study demonstrates that hUCB-MNCs administered intravenously at doses up to 8.57 × 10^8^ cells/kg do not cause adverse effects on fertility, embryo–fetal development, or postnatal offspring viability in Wistar rats. The absence of reproductive toxicity is mechanistically attributable to three intrinsic properties of hUCB-MNCs: their low immunogenicity, which prevents maternal immune activation; the protective function of the intact placental barrier; and their transient, paracrine-dominant mode of action, which limits exposure duration.

## 1. Introduction

The attention of the world scientific community has been focused on hUCB products for the treatment of various human diseases [1,2]. hUCB-MNCs contain a heterogeneous cell population, including hematopoietic stem cells (HSCs), endothelial progenitor cells (EPCs), mesenchymal stem cells (MSCs), unrestricted somatic stem cells (USSCs), lymphocytes, monocytes, etc. [3,4,5]. Their advantages, including non-invasive collection, low immunogenicity, multipotency, and a favorable safety profile regarding tumorigenicity, have spurred extensive research into their therapeutic potential for a wide range of conditions, such as cardiovascular diseases [6,7], neurological injuries [8,9], and perinatal ischemia [10,11]. Compared to human bone marrow and adipose-derived MSCs, hUCB-MNCs are easier to obtain, exhibit robust proliferation and differentiation capabilities, and demonstrate significant plasticity with minimal cell loss after cryopreservation [12,13,14,15].

Among hUCB-derived cells, MSCs have been the most extensively studied, with numerous preclinical and clinical trials demonstrating their efficacy and general biosafety [14,15,16,17,18,19,20,21,22,23,24]. However, hUCB contains a low percentage of mesenchymal stem cells, making it less suitable for clinical applications and necessitating the optimization of production methods. Mesenchymal stem cells isolated from perinatal tissue possess several properties essential for therapeutic use: they inhibit the proliferation of immune cells (T and B cells); induce the differentiation of macrophages toward an anti-inflammatory phenotype; stimulate tissue regeneration by paracrine effects (e.g., secretion of keratinocyte growth factor, hepatocyte growth factor, and epidermal growth factor); exert anti-inflammatory effects (suppression of interleukin-1β (IL-1β), tumor necrosis factor-α (TNF-α), and interleukin-8 (IL-8)); and promote vascular remodeling and other functions [25,26].

A less-studied area is the application of other mononuclear cells from umbilical cord blood (HSCs, EPCs, lymphocytes, monocytes) in the treatment of human diseases and their safety. The absolute numbers of T, B, and natural killer (NK) cells per unit volume, as well as the proportions of NK and B cells, are higher in umbilical cord blood than in peripheral blood. Studies have shown that NK cells can exert cytotoxic effects against breast and cervical cancer cells and produce high levels of IFN-γ and TNF-α when cultured in vitro [27]. Umbilical cord blood contains significantly more EPCs than peripheral blood or bone marrow. These EPCs play a crucial role in promoting the formation of stable and functional blood vessels, ensuring proper blood flow and selective permeability for macromolecules, as well as facilitating cytokine-induced leukocyte–endothelial interactions [27]. Due to their high therapeutic properties, human umbilical cord blood mononuclear cells are applied in preserving optic nerve cell function after injury [28,29], rapid recovery of the myocardium in a model of myocardial infarction [19], perinatal brain injury [30,31], and ischemia of various origins [32,33]. Additionally, hUCB-MNCs exhibit low immunogenicity due to the absence of certain antigens on their cell membranes, which can help prevent oncological transformation in recipient cells following transplantation. Their ability to produce various biologically active molecules—such as proteins with antioxidant properties, angiogenic factors, neurotrophic factors, and growth factors—further enhances their potential for stimulating regenerative processes in non-compatible recipients for a limited duration before being eliminated by the immune system [20,21,22,23,24]. A critical and underexplored aspect is the potential acute, chronic, reproductive and developmental toxicity of hUCB-MNCs. While studies on acute systemic toxicity [34,35], immunogenicity [36], and subchronic safety (90 days) [37,38,39] of umbilical cord-derived cells exist, they predominantly focus on purified mesenchymal stromal cell (MSC) populations and extracellular vesicles (EVs) isolated from hUCB-MNCs [40,41].

Data specifically dedicated to the heterogeneous hUCB-MNC product evaluated toxicity on fertility, embryonic, and fetal development are currently lacking. This gap is not merely academic; the unique composition of hUCB-MNCs, containing immunologically active lymphocytes and monocytes, necessitates a distinct safety assessment that cannot be fully extrapolated from studies on purified MSCs [34,35,36,37,38,39].

Consequently, the absence of robust preclinical data on reproductive toxicity constitutes a key barrier to the responsible clinical development of hUCB-MNC therapies for conditions affecting patients of reproductive age (e.g., perinatal injuries, autoimmune disorders, post-chemotherapy regeneration). Therefore, to directly address this unmet need and provide essential regulatory support, the present study was designed to conduct an investigation of the potential effects of intravenously administered hUCB-MNCs on critical reproductive endpoints in a standard rodent model.

Thus, the primary objective of this study was to perform a systematic study of the reproductive and developmental toxicity of intravenously administered hUCB-MNCs in a standard rodent model. The study was designed to assess potential adverse effects on critical endpoints, including fertility, early embryonic development, embryo–fetal development, and postnatal offspring viability.

## 2. Materials and Methods

### 2.1. Animals and Maintenance

The experiment was performed on Wistar rats obtained from the Laboratory Animal Nursery “Rappolovo,” a branch of the National Research Center “Kurchatov Institute.” The animals were maintained in a room with regulated temperature (21 ± 1 °C) and humidity (5–55%) with a 12-h light/dark cycle. During hUCB-MNCs injection, the rats were kept in cages (5 animals in each). Food and water were provided ad libitum. The main diet consisted of balanced pelleted feed. A total of 180 animals were used.

The study was conducted in accordance with the ethical standards established by the European Convention for the Protection of Vertebrate Animals Used for Experimental and Other Scientific Purposes (Strasbourg, 18 March 1986). This study was approved by the Clinical Research Ethics Committee of the N.V. Sklifosovsky Research Institute of Emergency Medicine University (protocol number 31; 19 June 2022). All umbilical cord samples were collected from healthy mothers after signing the informed consent.

### 2.2. hUCB-MNCs Administration

The samples of umbilical cord mononuclear blood cells were provided by the Sklifosovsky Research Institute for Emergency Medicine (Moscow, Russia). Before application, hUCB was resuscitated in a water bath at 37 °C and washed ex tempore with a transport medium (6 mL albumin (Kedrion, Gallicano, Italy) + 19 mL saline solution + 25 mL rheopolyglucine (Dextran, Minsk, Belarus)) to remove the cryomedium. Under sterile conditions, the blood sample was transferred into a 50 mL test tube and brought to the upper mark with sterile transport solution (the cell suspension was mixed thoroughly before administration). The cell suspension was stirred and centrifuged at +4 °C at 600× *g* for 10 min. The supernatant was discarded, and washing was repeated twice. Finally, the cell pellet was resuspended in a cell preservation solution (5% human albumin compound electrolyte solution) and further diluted to obtain the dose formulations with the required concentration according to the cell count results. All formulations were freshly prepared on the day of injection within 4 h. The concentrate of umbilical cord mononuclear blood cells was administered to female rats intravenously in the first half of the day as a cell suspension in a transport medium (0.5 mL per rat) using an insulin syringe with a non-removable needle (29 G (0.33 × 12)) by bolus injection [42,43,44].

The hUCB-MNCs product was characterized prior to administration. Cell viability, assessed by trypan blue exclusion, was consistently >90%. Flow cytometric analysis of the cell suspension was performed using a standard panel of antibodies to characterize the major cellular subpopulations. The median composition (*n* = 5 independent donor batches) was as follows: CD34+ hematopoietic stem/progenitor cells: 0.8–1.5%; CD3+ T lymphocytes: 50–65%; CD19+ B lymphocytes: 10–20%; CD56+ NK cells: 5–15%; CD14+ monocytes: 10–20%; and CD73+/CD90+/CD105+ MSCs: <1–2%. The product met pre-defined release criteria, including sterility (absence of microbial growth) and endotoxin levels (<0.5 EU/mL).

The hUCB-MNCs concentration was calculated based on the therapeutic dosage for humans (600 million cells per 70 kg of body weight (b.w.)) and was, respectively, 50 and 100 times the dose based on the animal’s body weight [44,45,46].

Dose per 1 kg = 600 × 10^6^ cells/70 kg ≈ 8.57 × 10^6^ cells/kg:

×50 dose calculation (low dose (LD)):

×50 = 8.57 × 10^6^ cells/kg × 50 = 428.5 × 10^6^ cells/kg = 4.28 × 10^8^ cells/kg.

×100 dose calculation (high dose (HD)):

×100 = 8.57 × 10^6^ cells/kg × 100 = 857 × 10^6^ cells/kg = 8.57 × 10^8^ cells/kg.

So, the low (×50) and high (×100) concentrations of the hUCB-MNCs were 4.28 × 10^8^ cells/kg and 8.57 × 10^8^ cells/kg, respectively.

The selected doses of hUCB-MNCs were determined based on previous acute toxicity studies. The low dose (4.28 × 10^8^ cells/kg) was selected to model a more realistic therapeutic exposure scenario. This dose-ranging approach, based on absolute cell counts, is a common and justified practice in preclinical safety assessment of cell-based therapies. Consequently, the high dose (8.57 × 10^8^ cells/kg) represents a substantial sub-lethal concentration, chosen to evaluate potential toxic effects under conditions of extreme exposure [44,47,48].

The animals were allowed to acclimatize for 14 days before the experimental study. After the end of the quarantine period, the animals were randomly assigned to statistical groups and weighed. At the end of the experiment (unless required by special experimental conditions), the animals were euthanized in a CO_2_ chamber.

### 2.3. Study Design

In the reproductive toxicity study, male and female Wistar rats (weighing 135–145 g, aged 12 weeks) were randomly assigned to three experimental groups (*n* = 30 per group; 10 males and 20 females): a negative control group (receiving an equivalent volume of transport medium), a low-dose group (4.28 × 10^8^ cells/kg), and a high-dose group (8.57 × 10^8^ cells/kg). To maintain a consistent male-to-female ratio of 1:2, each male rat was co-housed with two females from the same treatment group in a standard polycarbonate cage.

The administration of hUCB-MNCs to males was divided into two stages: on days 0 and 24. The introduction of males to intact females was carried out on days 49–50 from the moment of the first application of hUCB-MNCs (covering a complete spermatogenesis cycle).

In the developmental toxicity study, male and female rats (205–215 g, aged 12 weeks) were randomly divided into three groups (30 rats per group: 10 males and 20 females): a negative control group (administration of transport medium (TM) in equal volumes with animals in the experimental groups), a low dose group, and a high dose group. Males used for mating were not injected with hUCB-MNCs. Rats were grouped to maintain a consistent male-to-female ratio of 1:2. Only females with regular cycles were selected for the experiment. Before mating (twenty-two days), vaginal smears were taken from the females for microscopic examination to determine the phase of the estrous cycle (2–3 complete estrous cycles). The following parameters were identified: estrus (E), metestrus (M), diestrus (D), and proestrus (P). The day on which a vaginal plug was detected or a sperm-positive vaginal smear was confirmed was designated as Gestational Day (GD) 0.

The administration of hUCB-MNCs to female rats was divided into two stages: 14 days before mating and on the day of mating. This regimen was designed to ensure continuous exposure during the pre-maturation period of oocytes, the peri-conceptional interval, and throughout the subsequent gestation period following successful mating. On the GD20, the females were euthanized, and the abdominal cavity was opened. The uterus and ovaries were removed and placed on a Petri dish with saline. Fertility, pregnancy rate, corpora lutea, implantation sites, dead and resorbed fetuses, body weight, craniocaudal size of fetuses, and anomalies in fetal development were recorded for each pregnant female [43].

For detailed skeletal and visceral evaluations, a systematic random sampling strategy was employed. From each litter, approximately one-half of fetuses were randomly selected for skeletal examination, and another one-half were randomly selected for visceral examination, ensuring that no fetus was used for both analyses. This sampling approach resulted in a total of ≈50% fetuses per group for skeletal assessment and ≈50% fetuses per group for visceral assessment, distributed proportionally across all litters.

Developmental anomalies were recorded during the external examination of the fetuses. Some of the fetuses were fixed in Bouin’s solution (for at least 7 days), while the rest were fixed in 96% ethyl alcohol (5–7 days). Bouin’s fixative was prepared ex tempore from a saturated 1.2% aqueous solution of picric acid, concentrated formaldehyde (40%), and glacial acetic acid in a ratio of 15:5:1. The presence of developmental anomalies in the internal organs of fetuses was assessed using the cross-sectional method [49]. All examinations were conducted with a binocular magnifying glass MBS–10 (Micromed, Saint Petersburg, Russia).

Fetuses previously preserved in ethanol were placed in acetone for 18–20 h to remove fat-soluble structures. After defatting, the fetuses were transferred to a 2.75% aqueous solution of potassium hydroxide for 2–3 days at room temperature. The duration of contact with the alkali was determined by the degree of transparency of the skin and internal organs of the fetuses and was adjusted individually. Dawson’s staining with alizarin red S was then applied. The skeletal condition was evaluated using an operating surgical loupe. Four females from each group were selected for full-term birth and nursing of their offspring until weaning (26–30 days). The nature and duration of the birthing process were observed. Offspring standardization was not performed. In the growing offspring, the following parameters were recorded: mortality; developmental anomalies; weight gain; detachment of the auricle; appearance of hair; eruption of incisors; opening of the eyes; and, in males, descent of the testicles.

### 2.4. Statistical Analysis

GraphPad Prism 7 software was used to show all data reports (GraphPad, Inc., La Jolla, CA, USA). All data were assessed for normality of distribution using the Shapiro–Wilk test. The data are presented as the mean ± Standard Deviation (SD). Categorical data were presented as counts and percentages (*n*/N, %). A *p*-value of <0.05 was accepted as statistically significant.

For comparisons of a single continuous outcome across three independent groups (NC, LD, HD), one-way ANOVA was used, followed by Tukey’s post hoc test. To analyze the effects of two independent factors (e.g., Treatment Group and Time Point), two-way ANOVA was employed.

For categorical (frequency) data, such as fertility rates, pregnancy rates, and incidences of fetal anomalies, the Chi-square test with Yates’s continuity correction (for larger contingency tables) was used.

## 3. Results

### 3.1. Reproductive Toxicity Study

During the experimental study, no anomalies in animal behavior or in food and water consumption were detected. Clinical examinations of the animals did not reveal any physical abnormalities in male or female rats.

Throughout the mating period, no anomalies in sexual behavior were observed. Post-mating autopsies of males, conducted after 10 days, revealed no abnormalities in the topography, color, or size of internal organs, including the reproductive system.

Autopsies of females performed on GD20 showed normal conditions of internal organs in animals in the late stages of pregnancy, with no abnormalities found in the reproductive system.

Statistical evaluation of body weights before mating, during gestation, and lactation indicated a stable increase, except in cases of negative pregnancy (Table 1, Table 2 and Table 3), except for body weight gain during the lactation period (days 0–14), which was significantly lower in the high-dose group compared to the negative control (*p* = 0.02; Table 3).

No statistically significant differences were noted in gestational data such as fertility rate, pregnancy rate, corpora lutea counts, implantation sites, dead or resorbed fetuses, body weights, and craniocaudal sizes of fetuses across all dose groups compared to the negative control group (Table 4).

Examinations of external, visceral, and skeletal structures of the fetuses in all experimental groups revealed no abnormalities related. The skeletons, including the bones of the skull, thoracic region, and upper and lower limbs, exhibited a dark coloration, indicating normal ossification processes. No statistically significant differences were observed between groups for craniocaudal size (Table 5).

**Figure 1:**
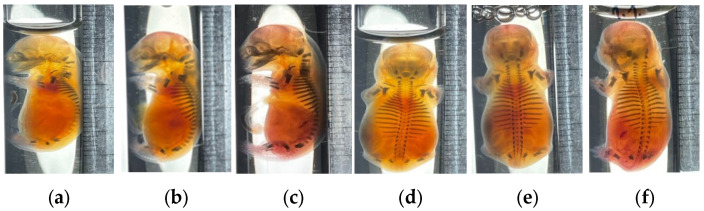
Skeleton of fetuses in fertility and early embryonic development toxicity assessment of human umbilical cord mononuclear blood cells: (**a**) negative control group; (**b**) low dose group (4.28 × 10^8^ cells/kg); (**c**) high dose group (8.57 × 10^8^ cells/kg); (**d**) negative control group; (**e**) low dose group (4.28 × 10^8^ cells/kg); (**f**) high dose group (8.57 × 10^8^ cells/kg). Panels (**a**–**c**) show lateral views; panels (**d**–**f**) show dorsal views.

No instances of fused, duplicated, displaced, incorrectly articulated, or missing vertebrae or ribs were observed. However, weak ossification (as indicated by staining intensity) of the proximal phalanges of the hind limbs was noted in fetuses from both control and experimental groups, which may be attributed to the developmental characteristics of this animal line (Figure 1a–f).

When analyzing the postnatal offspring development (F1), no significant differences in the anatomical features and behavior of the pups between the experimental and control groups were found.

The postnatal development of offspring from four randomly selected litters per group (NC, *n* = 58 pups; LD, *n* = 61 pups; HD, *n* = 54 pups) was monitored until weaning (day 26–30).

No offspring mortality was observed in any group during the entire lactation period. Furthermore, comprehensive daily clinical examinations revealed no instances of developmental anomalies, skin lesions (wounds, rash, redness), or tail defects in any offspring from the NC, LD, or HD groups.

### 3.2. Embryo–Fetal Development Study

During the experimental study, no anomalies in animal behavior or disturbances in food and water consumption were observed. Clinical examinations of the female rats revealed no physical abnormalities. Throughout the mating period, no irregularities in sexual behavior were detected.

Statistical evaluations indicated a stable increase in body weights before mating and during gestation, with the exception of cases of negative pregnancy (Table 6 and Table 7). No significant differences were found between groups at any time point.

Autopsies performed on females on GD20 of gestation showed normal conditions of internal organs in those at the late stages of pregnancy, with no anomalies found in the reproductive system. Similarly, females that did not become pregnant exhibited no reproductive system abnormalities.

No statistically significant changes were noted in the number of live females, gestational data (including fertility rates, corpora lutea counts, implantation sites, dead or absorbed fetuses), body weights, or fetal development anomalies across all dose groups compared to the control group. External, visceral, and skeletal examinations of the fetuses in all experimental groups revealed no findings related to the test article (Table 8 and Table 9).

In all experimental groups, the bones of the skull, thoracic region, and upper and lower extremities exhibited a dark coloration, indicating normal ossification processes. There were no instances of fused, duplicated, displaced, incorrectly articulated, or missing vertebrae or ribs. Additionally, no reduction in the number of ossification points in large and small bones was observed.

## 4. Discussion

This study provides the first assessment of the reproductive and developmental toxicity of human umbilical cord blood mononuclear cells (hUCB-MNCs) in a rodent model. The principal finding is that intravenous administration of hUCB-MNCs at doses up to 8.57 × 10^8^ cells/kg—representing a 100-fold multiple of the estimated human therapeutic dose—did not induce adverse effects on fertility, embryofetal development, or postnatal offspring viability in Wistar rats. Accordingly, the no-observed-adverse-effect level (NOAEL) for all reproductive and developmental parameters under the present experimental conditions is established at 8.57 × 10^8^ cells/kg.

The only statistically significant finding was a modest reduction in body weight gain among lactating dams in the high-dose group (Table 3). This effect, however, was not accompanied by any clinical signs of toxicity, did not affect offspring survival or development, and is considered biologically insignificant in the context of the established NOAEL for reproductive parameters.

The absence of external, visceral, and skeletal malformations in fetuses from hUCB-MNC-treated dams (Table 5 and Table 9; Figure 1) indicates that the developing conceptus experienced no adverse impacts under the conditions of this study. Although dedicated biodistribution studies were not performed—a limitation acknowledged below—the most plausible explanation is the functional integrity of the placental barrier. The size and phenotypic characteristics of hUCB-MNCs render passive transplacental migration across the intact syncytiotrophoblast layer highly improbable [50,51]. Furthermore, the lack of maternal toxicity (normal body weight gain and absence of clinical signs) suggests that no secondary inflammatory or metabolic disturbances capable of disrupting organogenesis were triggered. Postnatal monitoring of F1 offspring revealed no treatment-related mortality, growth retardation, or developmental anomalies, confirming the absence of latent or delayed adverse effects.

No statistically significant differences were observed between treated and control groups in fertility parameters, including mating success, fertility index, pregnancy rate, and the number of corpora lutea and implantation sites (Table 4). The absence of observable toxicity following administration of a heterogeneous allogeneic cell product can be explained by three complementary mechanisms:

First, hUCB-MNCs exhibit low immunogenicity, expressing minimal levels of major histocompatibility complex class II antigens and co-stimulatory molecules (e.g., CD40, CD80, CD86) [52,53,54]. This property minimizes the risk of eliciting robust adaptive immune responses or graft-versus-host-like reactions that could induce systemic inflammation and impair reproductive function [27,28,55,56]. The observed absence of maternal toxicity supports this interpretation.

Second, the placental barrier functions as a critical physical and immunological interface that effectively isolates the conceptus from circulating cells. While intravenously infused mesenchymal stromal cells (MSCs) may migrate to sites of injury or inflammation [56], their trafficking to healthy reproductive organs in the absence of chemotactic signals is inefficient. Given the cellular dimensions of hUCB-MNCs, substantial passive transmigration across an intact placenta is improbable. The absence of fetal anomalies, therefore, suggests effective fetal isolation from direct cellular exposure.

Third, hUCB-MNCs—particularly the MSC fraction—demonstrate transient persistence following allogeneic infusion, with rapid clearance by the recipient’s immune system [4,26]. Their primary therapeutic mechanism is paracrine signaling rather than long-term engraftment. Secreted factors (e.g., IL-10, TGF-β, HGF, PGE_2_) generally exert anti-inflammatory, pro-survival, and immunomodulatory effects [26,30,57]. In a healthy physiological context, this paracrine milieu is unlikely to disrupt the finely tuned hormonal and local immune environment required for ovulation, fertilization, and embryogenesis—contrasting with disease models where such signaling confers therapeutic benefit.

These findings align with and substantially extend the limited literature on umbilical cord-derived cell products. Li et al. [43] reported no reproductive toxicity following administration of purified human umbilical cord MSCs (hUC-MSCs) in rats. The present work advances this knowledge by demonstrating an equivalent safety profile for the heterogeneous, clinically relevant hUCB-MNC fraction, which contains immunologically active lymphocyte and monocyte populations. This distinction is critical for regulatory approval, which requires safety data generated with the final manufactured product rather than purified subpopulations. Importantly, the high dose evaluated (8.57 × 10^8^ cells/kg) substantially exceeds doses typically employed in efficacy studies [44,47], providing a robust margin of safety.

Several limitations warrant acknowledgment. First, although the rat model is regulatory-accepted for reproductive toxicity assessment, interspecies differences in placental structure, immune function, and cellular pharmacokinetics preclude direct extrapolation of absolute NOAEL values to humans. Second, skeletal and visceral examinations were performed on approximately 50% of fetuses, which, while consistent with OECD guidelines, may not capture extremely rare malformations. Third, the background malformation rate in the control colony was lower than the 1–2% typically reported in the literature, which may affect the sensitivity to detect small increases in developmental abnormalities. The absence of biodistribution data constitutes a gap in mechanistic understanding; future studies employing cell labeling or sensitive PCR-based detection methods are warranted to definitively quantify potential (if minimal) exposure of reproductive tissues or the fetus. Also, only two dose levels were evaluated; while this approach aligns with current guidance for cell-based product safety assessment, a full dose–response characterization would provide additional granularity.

## 5. Conclusions

This study provides the first assessment of the reproductive and developmental toxicity of human umbilical cord blood mononuclear cells (hUCB-MNCs) in a rodent model. The results demonstrate that intravenous administration of hUCB-MNCs at doses up to 8.57 × 10^8^ cells/kg did not induce adverse effects on fertility, embryo–fetal development, or postnatal offspring viability in Wistar rats under the present experimental conditions.

Based on these findings, the no-observed-adverse-effect level (NOAEL) for reproductive and developmental parameters is considered to be 8.57 × 10^8^ cells/kg—a dose equivalent to 100-fold the estimated human therapeutic dose, providing a substantial margin of safety. The absence of reproductive toxicity is mechanistically attributable to three intrinsic properties of hUCB-MNCs: their low immunogenicity, the protective function of the intact placental barrier, and their transient, paracrine-dominant mode of action.

Notwithstanding limitations of the study, mentioned in the discussion section, the consistent absence of treatment-related effects across multiple reproductive and developmental endpoints, together with the high dose multiples tested, supports the conclusion that hUCB-MNCs do not pose a significant reproductive or developmental hazard. These findings fill a critical regulatory gap by establishing the safety profile of the heterogeneous, clinically relevant hUCB-MNC product itself, rather than a purified subpopulation. Future studies incorporating biodistribution analysis and extended transgenerational assessments are warranted to further refine the safety characterization of this promising cell-based therapeutic.

## Figures and Tables

**Table 1:** Body weight of female rats during the pre-mating period (g).

	1 Week	2 Week	3 Week
NC	139 ± 18	146 ± 20	152 ± 22
LD	134 ± 14	141 ± 13	149 ± 17
HD	147 ± 17	153 ± 16	160 ± 16

Negative control group (NC) (administration of TM in equal volumes with animals of experimental groups); low (LD)- and high (HD)-dose groups. Data were analyzed using two-way ANOVA and Tukey’s multiple comparisons test, compared to the NC group, with *n* = 20 per group. The data are presented as the mean ± SD. No significant differences were found between groups at any time point (*p* > 0.05).

**Table 2:** Body weight gain of female rats of generation P (pregnancy) (g).

	0–7 Days	0–14 Days	0–20 Days
NC	32 ± 7	64 ± 8	142 ± 10
LD	33 ± 8	63 ± 11	139 ± 26
HD	30 ± 10	60 ± 14	139 ± 18

Negative control group (NC) (administration of TM in equal volumes with animals of experimental groups); low (LD)- and high (HD)-dose groups. Data were analyzed using two-way ANOVA and Tukey’s multiple comparisons test, compared to the NC group, with *n* = 20 per group. The data are presented as the mean ± SD. No significant differences were found between groups at any time point (*p* > 0.05).

**Table 3:** Body weight gain of female rats of generation P (lactation) (g).

	0–4 Days	0–7 Days	0–14 Days
NC	34 ± 15	37 ± 15	51 ± 17
LD	32 ± 16	36 ± 18	51 ± 19
HD	28 ± 18	29 ± 16	40 ± 17; * *p* = 0.02

Negative control group (NC) (administration of TM in equal volumes with animals of experimental groups); low (LD)- and high (HD)-dose groups, * *p* ≤ 0.05. data were analyzed by two-way ANOVA and Tukey’s multiple comparisons test, compared to the NC group, with *n* = 20 per group. The data are presented as the mean ± SD.

**Table 4:** Summary of gestational data in fertility and early embryonic development study (Generation P).

	NC	LD	HD
Fertility	90%	80%	95%
Pregnancy rate	100%	100%	100%
Corpora lutea count	13 ± 0.8	12 ± 0.5	13.5 ± 0.5
Implantation sites count	12 ± 0.9	11 ± 0.8	12 ± 0.7

Negative control group (NC) (administration of TM in equal volumes with animals of experimental groups); low (LD)- and high (HD)-dose groups. Implantation site counts and corpora lutea data were analyzed using one-way ANOVA followed by Tukey’s multiple comparisons test; fertility rate data were analyzed using the Chi-square test (with Yates’ correction). Fertility (%) = (number of pregnant females/number of females paired with males) × 100%; Pregnancy rate (%) = (number of females with live implants/number of pregnant females) × 100%. *n* = 20 in each group. The data are presented as the Mean ± SD. No significant differences were found between groups at any time point (*p* > 0.05).

**Table 5:** Summary of gestational data in fertility and early embryonic development study (Generation F1).

	NC	LD	HD
Live fetuses count in a generation	235/235 (100%)	205/205 (100%)	257/257 (100%)
Dead and resorbed fetuses	0/235 (0%)	0/205 (0%)	0/257 (0%).
Body weight (g)	5.4 ± 0.38	5.6 ± 0.29	5.38 ± 0.31
Craniocaudal size (mm)	4.5 ± 0.5	4.7 ± 0.4	4.9 ± 0.3
Skeleton deformity	0/120 (0%)	0/100 (0%)	0/130 (0%)
Visceral deformity	0/115 (0%)	0/105 (0%)	0/127 (0%)

Negative control group (NC) (administration of TM in equal volumes with animals of experimental groups); low (LD)- and high (HD)-dose groups. Craniocaudal size and body weight data were analyzed using one-way ANOVA. Craniocaudal size and skeletal deformity were measured in randomly selected fetuses, distributed across all litters: NC group—*n* = 120, LD group—*n* = 100, HD group—*n* = 130. Body weight was measured in all live fetuses (NC—*n* = 235, LD—*n* = 205, HD—*n* = 257). Body weight and craniocaudal size data are presented as the mean ± SD.

**Table 6:** Body weight of female rats during the pre-mating period (g).

	1 Week	2 Week	3 Week
NC	211 ± 22	220 ± 24	229 ± 25
LD	218 ± 32	231 ± 33	238± 34
HD	215 ± 21	224 ± 20	234 ± 21

Note: Negative control group (NC) (administration of TM in equal volumes with animals of experimental groups); low (LD)- and high (HD)-dose groups. Data were analyzed using two-way ANOVA followed by Tukey’s multiple comparisons test. *n* = 20 in each group. The data are presented as the mean ± SD. No significant differences were found between groups at any time point (*p* > 0.05).

**Table 7:** Body weight gain of female rats during the mating period (g).

	0–7 Days	0–14 Days	0–20 Days
NC	31 ± 6	63 ± 7	137 ± 16
LD	33 ± 9	68 ± 11	147 ± 19
HD	29 ± 9	62 ± 11	134 ± 21

Note: Negative control group (NC) (administration of TM in equal volumes with animals of experimental groups); low (LD)- and high (HD)-dose groups; data were analyzed using two-way ANOVA followed by Tukey’s multiple comparisons test. No significant differences were found between groups at any time point (*p* > 0.05). *n* = 19 in each group. The data are presented as the mean ± SD. No significant differences were found between groups at any time point (*p* > 0.05).

**Table 8:** Gestational and fetal data from the pregnant female embryo–fetal development study (generation P).

	NC	LD	HD
Live females count	20/20 (100%)	20/20 (100%)	20/20 (100%)
Fertility	95%	95%	90%
Corpora lutea count	13 ± 2.5	15 ± 1.8	14.3 ± 1.4
Implantation sites	12.1 ± 2.4	12.9 ± 3.3	12.06 ± 2.8

Negative control group (NC) (administration of TM in equal volumes with animals of experimental groups); low (LD)- and high (HD)-dose groups. For implantation sites count, corpora lutea count data were analyzed using one-way ANOVA followed by Tukey’s multiple comparisons test; fertility data were analyzed using Chi-square test (with Yates’ correction). *n* = 19 in each group. The data are presented as the mean ± SD. Fertility (%) = (number of pregnant females/number of females paired with males) × 100%.

**Table 9:** Gestational and fetal data from the pregnant female embryo–fetal development study (generation F1).

	NC	LD	HD
Dead and resorbed fetuses	3/228 (1.32%)	3/253 (1.19%)	1/230 (0.43%)
Body weight (male)	6.7 ± 2.6 *n* = 108	6.5 ± 1.5 *n* = 123	6.6 ± 1.5 *n* = 120
Body weight (female)	6.2 ± 2.8 *n* = 117	6.7 ± 1.9 *n* = 127	6.05 ± 2.5 *n* = 109
The male/female ratio	0.480	0.492	0.524
Fetal development anomalies	0/225	0/250	0/229
Skeleton deformity	0/110 (0%)	0/125 (0%)	0/110 (0%)
Visceral deformity	0/110 (0%)	0/125 (0%)	0/110 (0%)

Note: Negative control group (NC) (administration of TM in equal volumes with animals of experimental groups); low (LD)- and high (HD)-dose groups. Body weight data were analyzed using one-way ANOVA followed by Tukey’s multiple comparisons test; dead and resorbed fetuses’ data were analyzed using Chi-square test (with Yates’ correction). Body weight data are presented as the mean ± SD.

## Data Availability

The original contributions presented in this study are included in the article. Further inquiries can be directed to the corresponding authors.

## Abbreviations

The following abbreviations are used in this manuscript:

D	Diestrus
E	Estrus
EGF	Epidermal growth factor
EPCs	Endothelial progenitor cells
HGF	Hepatocyte growth factor
HSCs	Hematopoietic stem cells
hUCB	Human umbilical cord blood cell
hUCB-MNCs	Human umbilical cord mononuclear blood cells
IL-10	Interleukin-10
IL-1β	Interleukin-1β
IL-4	Interleukin-4
IL-8	Interleukin-8
KGF	Keratinocyte growth factor
M	Metestrus
MSCs	Mesenchymal stem cells
NK	Natural killer
NOAEL	No-observed-adverse-effect level
OECD	Organization for Economic Co-operation and Development
P	Proestrus
TM	Transport medium
TNF-α	Tumor necrosis factor-α
USSCs	Unrestricted somatic stem cells
